# Culturally Optimised Nutritionally Adequate Food Baskets for Dietary Guidelines for Minimum Wage Estonian Families

**DOI:** 10.3390/nu12092613

**Published:** 2020-08-27

**Authors:** Janne Lauk, Eha Nurk, Aileen Robertson, Alexandr Parlesak

**Affiliations:** 1 Clinical Research Centre, Department of Clinical Sciences, Faculty of Medicine, Lund University, Jan Waldenströms gata 35, 214 28 Malmö, Sweden; lauk.janne@gmail.com; 2 Global Nutrition and Health, University College Copenhagen, Sigurdsgade 26, 2200 Copenhagen, Denmark; aileen.robertson@foodconsult.info; 3 Department of Nutrition Research, National Institute for Health Development, Hiiu 42, 11619 Tallinn, Estonia; eha.nurk@tai.ee

**Keywords:** nutritionally adequate diet, linear programming, cultural acceptability, affordable diet, low socioeconomic status, food-based dietary guidelines (FBDG)

## Abstract

Although low socioeconomic groups have the highest risk of noncommunicable diseases in Estonia, national dietary guidelines and nutrition recommendations do not consider affordability. This study aims to help develop nutritionally adequate, health-promoting, and culturally acceptable dietary guidelines at an affordable price. Three food baskets (FBs) were optimised using linear programming to meet recommended nutrient intakes (RNIs), or Estonian dietary guidelines, or both. In total, 6255 prices of 422 foods were collected. The Estonian National Dietary Survey (ENDS) provided a proxy for cultural acceptability. Food baskets for a family of four, earning minimum wage, contain between 73 and 96 foods and cost between 10.66 and 10.92 EUR per day. The nutritionally adequate FB that does not follow Estonian dietary guidelines deviates the least (26% on average) from ENDS but contains twice the sugar, sweets, and savoury snacks recommended. The health-promoting FB (40% deviation) contains a limited amount of sugar, sweets, and savoury snacks. However, values for vitamin D, iodine, iron, and folate are low compared with RNIs, as is calcium for women of reproductive age. When both the RNIs and dietary guidelines are enforced, the average deviation (73%) and cost (10.92 EUR) are highest. The composition of these FBs can help guide the development of dietary guidelines for low income families in Estonia.

## 1. Introduction

In Estonia in 2018, the top three causes of mortality were the preventable noncommunicable diseases (NCD): ischemic heart disease, hypertensive heart disease, and stroke [1]. Among the most important behavioural risk factors for cardiovascular diseases is an unhealthy diet [2], which includes a high consumption of saturated fats, salt, and refined carbohydrates, as well as low consumption of fruits and vegetables [3]. It is reported that Estonians consume barely two portions of vegetables per day (1.6–2.0 for men and 1.8–2.5 for women) [4,5] compared with the recommended 7–10 and 6–8, respectively [6]. The median vegetable and fruit intake is less than half (<200 g/day) the recommended intake of >400 g and less than half the population (48%) reported consuming vegetables daily [6]. In Europe, Estonia has among the lowest proportion of adolescents consuming vegetables (24%) and fruits (35%) daily and their consumption decreases with increasing age from 11 to 15 years of age [7]. In the total population, the intake of salt, sugar-sweetened beverages, and trans-fats is around double that recommended. Nearly half (47.4%) of the population consume too much total fats [8] compared with the national food-based dietary guidelines (FBDG) [6]. Over four-fifths (84.3%) of the population consume too much saturated fats and just over half (53.3%) do not eat enough fibre [8]. These unhealthy dietary practices contribute to the high prevalence of overweight and obesity: 32% and 19% in adults (16–64 years) [9] and 14% and 3% in adolescents (11–15 years) [10], respectively. Twenty-one percent of Estonian women and 34% of men have raised blood pressure [11] and those most at risk of developing CVD are those with low SES [12]. There is, therefore, a pressing need for low SES families to replace high-fat energy dense foods with foods rich in complex carbohydrates and dietary fibre, including vegetables and fruits.

High socioeconomic families eat more vegetables and fruits than low socioeconomic families [13]. Accordingly, Estonian health outcomes are worsening with increasing social disadvantages in income, education, social position, and employment [14]. For example, Estonians with the lowest compared with the highest level of education are 40% more likely to suffer from hypertension, and levels of obesity are over 20% higher in the less compared with the well-educated (22% versus 18%) [15]. Estonia is one of the EU countries with the greatest, and increasing, inequalities in health [16].

Several social protection measures, such as unemployment benefits, have been put in place by the Estonian government in order to attempt to reduce disparities. For example, the government has increased the minimum wage, the tax-free minimum on earnings, and the child allowance support. However, despite these social benefits, the lowest quintile still receives an income that is more than six times lower than the top quintile of the population. In 2018, at least half of the unemployed were at risk of poverty: on average, one-fifth (22%) of the population lived in relative poverty and about two percent (2.4%) in absolute poverty [17,18,19].

The main goal of this study is to compile lists of locally available foods (food baskets, FBs) that are nutritionally adequate, health-promoting, culturally acceptable, and affordable for a low income Estonian family of four. A well-established method to achieve nutritional adequacy (i.e., cover all acceptable macronutrient distributions (AMDR) and recommended nutrient intakes (RNIs)) is linear optimisation or linear programming (LP). Linear programming can provide dietary solutions that meet RNIs alone, food-based dietary guidelines (FBDG) alone, or both these constraints together, while being optimised for cultural acceptability [20,21,22,23]. Once compiled, these FBs could form the basis of Estonian FBDG for low income families and subsequently help to guide the reduction of health inequalities related to diet-associated NCD.

## 2. Materials and Methods

### 2.1. Foods, Their Prices, and Their Categorisation

In total, 422 foods and 6255 online prices were collected from three different supermarket chains’ websites [24,25,26] during May and June 2017. Data (name, weight, and price) were collected for raw/uncooked foods and ready-to-eat products. For all items, the price per kilogram or litre was calculated.

The foods, with their generic names along with their median specific prices, were arranged into categories as defined in the Estonian National Dietary Survey (ENDS) [27,28,29]. The six main categories, or food groups, are: “Starchy foods—Cereals and potatoes”; “Fruits, vegetables and berries”; “Milk and dairy products”; “Fish, poultry, eggs, meat and meat products”; “Added fats, nuts, seeds and oleaginous fruits”; and “Sugar, sweets and savoury snacks”. These were further subdivided into 48 subcategories as outlined in the Estonian survey [5].

### 2.2. Nutritional Composition

Nutritional composition was collected from various databases. The priority in which these databases were used (in the order of the highest priority to the least) was: NutriData, an Estonian food composition database [30]; Fineli, the national food composition database in Finland [31]; Fødevaredatabanken, a Danish food composition database [32]; a Swedish food composition database [33]; Matvaretabellen, a Norwegian food composition database [34]; McCance and Widdowson’s ‘composition of foods integrated dataset’ on the nutrient content of the UK food supply [35]; and the United States Department of Agriculture (USDA) National nutrient database for standard reference [36]. If foods were consumed after preparation, the nutrient composition of the prepared (cooked, simmered, baked etc.) food was used for calculations. In addition to the values for the nutrients, the fibre and water (for both raw and prepared foods), along with the value of the edible proportion, were recorded. The bioavailability of micronutrients in foods, the yield factors (weight changes during preparation), and the size of the edible proportions were calculated as described previously [22,37].

### 2.3. Nutritional Adequacy

Estonian RNIs values are used as the reference for a nutritionally adequate diet [6] in both the lowest cost FB (LCFB) and the nutritionally adequate diet FB (NFB). The RNIs values not available in the Estonian dietary guidelines (such as those on ω-6 fatty acids) were taken from the World Health Organization [3]. The national dietary guidelines provided the estimated energy requirement (EER) for each family member along with the acceptable macronutrient distribution ranges (AMDR) expressed as percentages of EER: 10–20% protein; 25–35% lipids; max 10% saturated fatty acids; 10–20% monounsaturated fatty acids; 5–10% polyunsaturated fatty acids; min 1% ω-3 fatty acids; max 1% trans-fatty acids; 50–60% carbohydrates; <5% added sugars [3,6]. The values for average body weight, physical activity levels (PAL), EER, and RNIs for each family member are shown in Table 1.

### 2.4. An Average Estonian Household

An average household composition was modelled using data from the Population and Housing Census for Estonia 2014 [38]: male (father) aged 40–44 years; female (mother) aged 35–39 years, and two adolescents (15 year old daughter and 13 year old son). Linear optimisation was run individually for each family member and the resulting food baskets were combined to give the total daily FB for the household.

### 2.5. Food-Based Dietary Guidelines (FBDG)

The intake amounts of food groups recommended in the national dietary guidelines [6] were included in some of the optimisations to enforce a health-promoting diet in both the health promoting FB (HFB) and the nutritionally adequate and health promoting FB (NHFB). The number of daily food portions recommended in the national dietary guidelines is shown in Table 2 and is based on the EER for each family member. The weight of food portion sizes as recommended in the national guidelines was used, for example, the weight of one portion of bread per day is 30 g. Where Estonian portion sizes were not available, the medium daily portion sizes listed in the Finnish food composition database were used [31].

### 2.6. Estonian Minimum Monthly Wage

From 1st January 2018, the Estonian minimum monthly wage per capita was 500 EUR, which provides a net income of 482 EUR per month [39]. The amount of the household budget available to buy food and drinks was calculated by assuming both adults earned the same minimum wage. In addition, an Estonian family of four received social benefit allowances for each child (55 EUR per child and month) amounting to a total family income of 1074 EUR per month. According to the Household Budget Survey, 2016 households from the lowest income quintile spend on average 27.8% of their income on food and non-alcoholic beverages, and 2.4% on alcoholic beverages [40]. The following formula was used to calculate the budget available for food and beverages (Minimum Wage Food Cost, *MWFC*) per day.

(1) $$\begin{array}{c} MWFC=\frac{[\left(2\times \;Parent^{\prime }s\;min.\;wage+2\;\times \;Child\;benefit\right)\;\times \;12\;months/year\;\times \;share\;of\;income\;spent\;on\;food\;\&\;NA\;beverages}{Number\;of\;days\;per\;year}\\ =\;\frac{\left(2\;\times \;482\;€+2\;\times \;55\;€\right)\;\times \;12\;\times \;0.302\;}{365.25}\;\approx €10.66 \end{array}$$

### 2.7. Culturally Acceptable Food for Estonian Households

Data from the ENDS are used to represent current eating habits and cultural acceptability by sex and age group for each family member [5]. Cultural acceptability of the FBs is achieved by minimising the deviation from the ENDS. To assess the similarity between the ENDS and the FBs, the absolute (non-negative) value of the relative deviation [*abs*(*RD*)] was calculated for each of the 45 food subcategories.

(2) $$abs\left(RD_{i}\right)=\frac{abs\left(m_{i}-M_{i}\right)}{m_{i}}$$

In Equation (2), *m* stands for the observed weight of the *i*-th food category (grams) after optimisation and *M_i_* is the weight of the *i*-th food category consumed by the corresponding family member [29]. In order to minimise deviation from the ENDS, the total relative deviation (*TRD*) was calculated as the sum of *abs(RD_i_)* for all foods in the FBs (Equation (3)).

(3) $$TRD\;=\;\sum_{i=1}^{n}abs\left(RD_{i}\right)$$

The minimum of *TRD* was used as the objective function in three FBS (NFB, HFB, and NHFB). The average relative deviation (*ARD*, Equation (4)) of food baskets from the ENDS is a proxy for how different the FBs were from the actual food intake (cultural acceptability).

(4) ARD=TRD/n

### 2.8. Linear Programming

Linear programming (LP) is a mathematical method for the minimisation or maximisation of a given linear goal (objective) function subjected to a set of constraints on a list of decision variables [41]. The underlying algorithm builds on three major elements: (i) the objective function, which is a loss function or its negative of the goal variable; (ii) the decision variables of the model, which are the amounts of foods to be included in the optimised FBs; (iii) a set of constraints (criteria to be met). Constraints can be applied to the model by defining minimum or maximum thresholds e.g., cost, nutrients, or the minimum or maximum weights of food groups recommended within national or international FBDG. If the algorithm calculates a model that meets all applied constraints, then, a feasible solution is said to be found [41]. In LP models, constraints that determine the extent to which the objective function (here, nutrient constraints) can be minimised or maximised are called “active constraints” [23,42,43]. Linear optimisation was done with the COIN-OR CBC optimisation engine algorithm, which is part of the open source add-in OpenSolver (v. 2.9.0) for MS Excel^®^ [44].

### 2.9. Dietary Diversity

The LP methodology allows the calculation of a lowest cost food basket (LCFB) that covers all RNIs, but where very few foods are selected. This means that unless the LP methodology is modified, the dietary diversity in the food baskets is very limited and not culturally acceptable [22]. In order to overcome this, a minimum number of different foods, especially vegetables, fruits, and cereals, are enforced. LP was forced to select a minimum of two varieties from food groups that contained between 15 to 19 food items (e.g., fresh meat or cheese); a minimum of three varieties from food groups that contained between 20 to 24 food items (no food groups); and a minimum of four varieties from food groups that contained more than 24 food items (e.g., vegetables and fruits).

### 2.10. Constraints Enforced for Each Food Baskets

Four FBs are constructed to compare their differences in cost and nutritional adequacy: (i) LCFB—lowest cost food basket; (ii) NFB—nutritionally adequate food basket; (iii) HFB—health-promoting food basket; (iv) NHFB—nutritionally adequate and health promoting food basket. Their specific objective function and the enforced constraints are shown in Table 3. The number and weight of foods are calculated for each FB. In order to construct average monthly FBs for one household, the daily amounts were multiplied by 30.4 (average number of days per month).

#### 2.10.1. The Lowest Cost Food Basket (LCFB)

The LCFB is calculated to find the absolute lowest cost of a FB that fulfils all Estonian nutrient recommendations (EER, AMDR, and RNIs) [6], using the minimum cost as goal function, but does not consider dietary diversity or cultural acceptability. The share of the budget for each family member is calculated based on cost proportions found in the LCFB.

#### 2.10.2. Nutritionally Adequate Food Basket (NFB)

The NFB is calculated by enforcing the Estonian nutrient recommendations (EER, AMDR, RNIs) and the minimum wage cost constraint as applied to the LCFB. Instead of the lowest cost, the NFB uses the least deviation from reported food subgroup intakes as the goal function (= minimum total, TRD) as reported in the Estonian National Dietary Survey [29].

#### 2.10.3. Health-Promoting Food Basket (HFB)

The HFB is also calculated using the minimum wage cost constraint to meet all EER, but not the AMDR or RNIs. Instead, the portion-based FBDG provided in the Estonian dietary guidelines [6] are enforced. These include the number of portions from six main food groups and ten subgroups required by each family member to fulfil the national dietary guidelines (Table 2). As the HFB is not constrained by AMDR or RNIs, this calculation helps to investigate how well the national dietary guidelines fulfil the AMDR and RNIs for a low income family. In addition, the goal function minimises the TRD from the ENDS.

#### 2.10.4. Nutritionally Adequate and Health-Promoting Food Basket (NHFB)

The NHFB is calculated to combine the constraints from both the NFB (enforced EER, AMDR, and RNIs) and the HFB (enforced portion-based FBDG) within the minimum wage cost constraint. In addition, the least possible TRD from the ENDS is used as goal function.

## 3. Results

### 3.1. The Lowest Cost Food Basket (LCFB)

The cost of the LCFB for a family of four is 4.11 EUR per day. This most cost-efficient food basket contains only nine foods but fulfils all the RNIs [6] for all family members (Table 4). The contribution of each food needed to cover the AMDR and RNIs is listed in Table S1. The ARD from the ENDS is 415% (Table 4).

### 3.2. Nutritionally Adequate Food Basket (NFB)

The NFB, which covers all the RNIs, is optimised to be as similar as possible to the ENDS and contains 96 foods and costs 10.66 EUR per day (Table 5). The ARD from the ENDS is about 26%. Around three-quarters (77%) of the total weight come from three food groups: “Starchy foods—Cereal and potatoes”, “Fruits vegetables and berries”, and “Milk and dairy products”. The two most expensive food groups (25% and 24% respectively of total cost) are “Fish, poultry, eggs, meat and meat products” at 2.69 EUR per day and “Starchy foods—Cereals and potatoes” at 2.57 EUR per day (Table 5); the least expensive (2% of total cost) is “Added fats, nuts, seeds and oleaginous fruits” at 0.23 EUR per day (oils/fats at 0.16 EUR and nuts/seeds at 0.07 EUR (3.9 g peanuts, 6 g sunflower seeds, and 7.4 g linseeds)). The NFB contains 11.5 portions of vegetables and fruits per family and day and 33.7 portions of sugar, sweets, and savoury snacks for the entire family (Table 5). Information about the composition of the monthly NFB is presented in Table S2.

### 3.3. Health Promoting Food Basket (HFB)

The HFB is optimised to be as similar as possible to the ENDS and is constrained to meet the Estonian dietary guidelines, but not the RNIs. The HFB contains 73 foods and costs 10.66 EUR per day (Table 5). The ARD from the ENDS is ~40%. Around four-fifths (81%) of the total weight come from three food groups: “Fruits, vegetables and berries”; “Starchy foods—Cereals and potatoes”; and “Milk and dairy products”. The most expensive food group (25% of total cost) is “Fruits, vegetables and berries” which costs 2.69 EUR per day; the least expensive (5% of total cost—2.5% each, oils/fats and nuts/seeds) is “Added fats, nuts, seeds and oleaginous fruits” which costs 0.51 per day EUR (0.26 EUR each for oils/fats and nuts/seeds (52 g peanuts and 18 g linseed seeds)). The HFB contains 26 portions of vegetables and fruits per family and day along with 16 portions of sugar, sweets, and savoury snacks (Table 5).

As the HFB is not constrained by complying with RNIs, its nutrient composition was investigated and compared with RNIs. The proportion of vitamin D and iodine is below the RNIs for all family members: mother (66% and 69%, respectively); girl (79% and 78%, respectively); father (74% and 83%, respectively); boy (72% and 81%, respectively) (Table 6). The proportion of iron, folate, and calcium is below the RNIs for the mother (81%, 78%, and 87%, respectively). The percentage of saturated fats and sodium are above the recommended levels for all family members. The percentage of total lipids are well above the recommended level for all except the mother (<110%) (Table 6). In addition, the percentage of total added sugar is above that recommended for the girl (323%), the mother, and the son (both 128%). The composition of the monthly HFB is presented in Table S3.

### 3.4. Nutritionally Adequate and Health-Promoting Food Basket (NHFB)

The NHFB, which covers both the RNIs and portion-based FBDG, is also optimised to be as similar as possible to the ENDS. The NHFB contains 92 foods and costs 10.92 EUR per day (Table 5). The ARD from the ENDS is ~73%. More than four-fifths (84%) of the total weight come from three food groups: “Starchy foods—Cereals and potatoes”, “Fruits, vegetables and berries”, and “Milk and dairy products”. The most expensive food group (27% of total cost) is “Fruits, vegetables and berries”, which costs of 2.90 EUR per day; the two least expensive are “Sugar, sweets and savoury snacks” with 9% of total at a cost of 0.97 EUR and “Added, nuts, seeds and oleaginous fruits” 5% of total (2.5% oils/fats and 2.5% nuts/seeds) at a cost of 0.54 EUR (0.27 EUR oils/fats and 0.27 EUR nuts/seeds) per day. The NHFB, similarly to the HFB, contains 26 portions of vegetables and fruits per day and 16 portions of sugar, sweets, and savoury snacks (Table 5). A detailed composition of the monthly NHFB is presented in Table S4.

**Figure 1:**
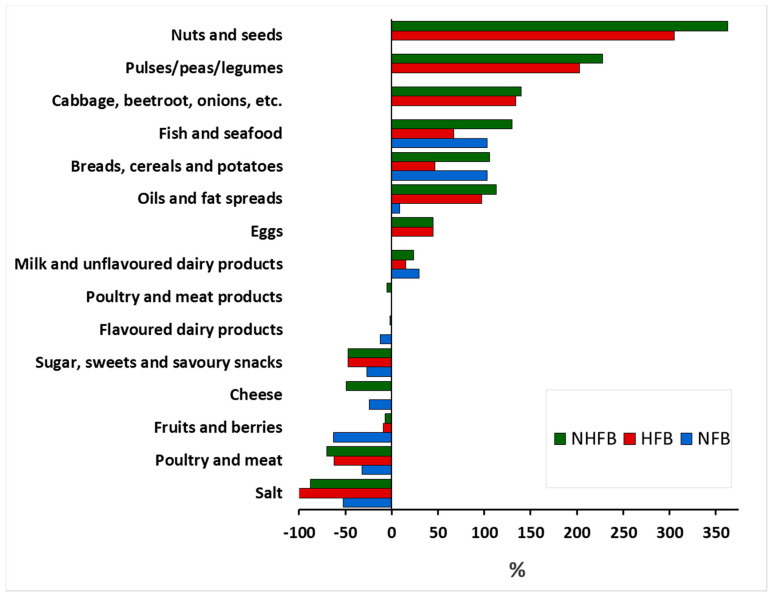
The relative differences between the contents of nutritionally adequate food basket (NFB, blue columns), health promoting food basket (HFB, red columns) and the nutritionally adequate and health promoting food basket (NHFB, green columns) and the Estonian National Dietary Survey (ENDS). The costs of the food baskets include the cost of alcoholic beverages.

The relative differences between the contents of the NFB, HFB, NHFB, and the ENDS are presented in Figure 1. The food groups not consumed in sufficient quantities to meet the recommendations are (in order of largest to smallest amounts): nuts and seeds; pulses, peas, and legumes; vegetables (e.g., cabbage, marrow, onion, spinach, beets, mushrooms); fish and seafood; oils; and bread, cereals, and potatoes. In contrast, salt, meat, cheese, and sugar, sweets, and savoury snacks are eaten in excess of the recommendations.

## 4. Discussion

When optimising for cultural acceptability and constraining for only RNIs (without FBDG), the NFB achieved a nutritionally adequate diet for an Estonian family of four living on the minimum wage. However, the NFB contains double the amount of sugar, sweets, and savoury snacks recommended in the Estonian FBDG. When these FBDG (without RNIs) are applied while optimising for cultural acceptability, the food basket (HFB) contains inadequate amounts of several nutrients such as vitamin D. The HNFB (with both RNIs and FBDGs) cost negligibly more (0.26 EUR per day) than the NFB and the HFB, but it meets both the nutrient recommendations and the dietary guidelines, although it deviates most from cultural acceptability. All optimised FBs have, compared to the food intake reported by the ENDS, a lower content of sugar, snacks, poultry, and meat.

### 4.1. Lowest Cost Food Basket (LCFB)

The LCFB fulfils all the recommended nutrient intakes for the lowest possible price (4.11 EUR) [6]. However, its contents are inadequate in terms of dietary diversity and cultural acceptability as it contains only nine foods (Table 4). In addition, it deviates on average by more than four hundred percent (415%) from the ENDS (Table 4), which provides a proxy for cultural acceptability. Clearly, the lowest cost FB is inadequate to form the basis for dietary guidelines for low income families. A recent review outlines weaknesses of the LP methodology and these include the tendency to select the cheapest but limited number of foods. It is, therefore, recommended that LP methods are adapted to consider both cultural acceptability and dietary diversity [45,46]. Such methodological adaptations were incorporated in the FBs below.

### 4.2. Nutritionally Adequate Food Basket (NFB)

The NFB, similar to the LCFB, covers the RNIs, but was optimised to be as similar as possible to the ENDS in order to increase cultural acceptability. The NFB is the FB that is most similar to the ENDS (ARD is only 26%), and therefore, most culturally acceptable. Its dietary diversity is improved to 96 foods from only nine (in the LCFB) but it still contains less than half the recommended amount of vegetables and fruits and contains double the amount of sugar, sweets, and savoury snacks (33.7 portions) compared with the upper tolerable limit (16 portions). The authors of the IDEFICS study in 2014 showed that a ‘processed’ eating pattern (high intakes of crisps, candy bars, savoury pastries, biscuits and packaged cakes, and sweetened drinks) is significantly inversely associated with low SES families in Estonia. Moreover, a ‘healthy’ pattern (fruits and vegetables, less fried foods, nuts, seeds, and dried fruits) was positively associated with high SES [47]. As discussed earlier, the prevalence of obesity is high among the low SES families and the inequality gap has increased three-fold in 15 years [7]. Given the high average prevalence of obesity in low SES Estonian families [48,49], the average EER values enforced in this study are probably too high to support healthy weight changes needed in low income families [50]. If low income families could be persuaded to substitute some of their meat and poultry purchases with legumes and pulses, this could increase their fibre and vegetable intake and reduce the amount they spend on food.

### 4.3. Health-Promoting Food Basket (HFB)

The cost of the HFB and NFB are the same (10.66 EUR per day). The HFB contains less unhealthy snacks compared with NFB. However, the HFB deviates from the ENDS by 40% compared with only 26% in the NFB. Instead, the HFB contains less sugar, sweets, and savoury snacks along with the recommended number of portions of vegetables and fruits (26 portions per day). One-third (30%) of the family’s actual food budget is spent on sugar, sweets, and savoury snacks compared with the HFB (where the cost would be <10%).

Although most of the RNIs are reasonably well covered in the HFB, several important nutrients are too low. For example, as shown in Table 6, the HFB has difficulty in reaching the RNIs for vitamin D and iodine for all family members, especially for the mother, whose values reach only two-thirds of her RNIs. The mother’s iron, folate, and calcium values are also below the RNIs. Specific recommendations are needed for low income Estonian families to ensure that their intakes of vitamin D and iodine are covered. For example, if the intake of tinned oily fish, such as sprats and cod roe, is increased, this will help meet both vitamin D and fish intake recommendations at minimal cost.

Mild iodine deficiency in Estonia could be eradicated through the implementation of universal salt iodisation (USI) [51]. USI enables governments to achieve the two public health goals of increasing iodine and reducing salt supply for the whole population [52]. However, USI has not been adopted into Estonian legislation [11] and this should be recommended as an integral part of the dietary guidelines for low income families. Given the prevalence of anaemia (25%) in Estonian women and girls [11], combined with a high prevalence of overweight/obesity [53], specific recommendations are needed. Low SES families should be recommended to buy low cost foods that are rich in nutrients (iron, folate, and calcium) but relatively low in energy.

### 4.4. Nutritionally Adequate, Health-Promoting Food Basket (NHFB)

The NHFB is optimised to meet both the RNIs and the Estonian FBDG along with being as similar as possible to the Estonian food intake. The NHFB contains 92 foods and costs 0.26 EUR/day more than the HFB or NFB (Table 5). If this reaches the limit of affordability for low income families, additional financial support should be available via government policies. In addition, because the NHFB deviates most from the Estonian food intake, low SES families will have to change their eating habits quite considerably. The NHFB has the best dietary diversity as it contains 23 varieties of vegetables and fruits (11 different fruits and 12 different vegetables, including pulses) in the recommended 26 portions (>2 kg) per day and contains the lowest amount of sugar and sweets. Estonian children have among the highest energy intake (380 kcal per day) from sugar sweetened beverages (SSBs) in Europe, which adversely affects cardiovascular disease risk [54]. SSBs appear to be an important predictor of families’ dietary patterns, where parents and children share a liking for sweet and fatty foods and beverages [55].

In Estonia, the availability of vegetables and fruits (not including potatoes and roots) is only 518 g/capita per day [56]. This falls short of the recommended availability of 800 g/capita per day [57] to ensure an intake of at least 400 g per person per day. Estonian food-based dietary guidelines for low SES groups should, therefore, consider the links between food availability, eating patterns, and social vulnerabilities in order to successfully reduce the health inequalities related to NCD [58] and also be included in Estonia’s new strategy [59] to achieve the sustainable development goals [60,61].

Using the information derived from the LP optimisation process carried out on these different food baskets, it is recommended that Estonian FBDG for low SES families should include the following recommendations (Table 7).

## 5. Limitations

The cost of the calculated FBs only applies to the purchase of food and it does not include expenses associated with food storage (cooling, freezing) and food preparation such as cooking, equipment, and time. The consumption of alcohol, and the necessary reduction, are not discussed within this paper despite being a major risk factor for Estonians’ health outcomes. Additionally, the FBs are designed for a reference Estonian family of four and do neither apply to people outside the reference age ranges, outside the normal weight reference range (BMI 18.5–25 kg/m^2^), nor to individuals with special nutritional needs, such as pregnant or lactating women, and people with food intolerance or allergy. Cost linked to food waste or foods that spoil after purchase are not considered. The prices of foods used in this study were collected online and may differ from prices in markets and at other retailers. However, as the online trading of foods has to stay competitive compared with over-the-counter selling, major differences in food prices in 2017 are unlikely.

## 6. Conclusions

The main goal of this study is to compile lists of locally available foods (food baskets, FBs) that are nutritionally adequate, health-promoting, culturally acceptable, and affordable for a low income Estonian family of four. These FBs could help create Estonian FBDG for low income families. The HNFB (with both RNIs and FBDGs) costs negligibly more (0.26 EUR per day) than the NFB and the HFB, but it includes both the nutrient recommendations and the food-based dietary guidelines. However, it deviates most from Estonian food intake and may be less culturally acceptable. Based on the overall analysis of food baskets, specific guidelines are presented in order to reduce the risk of both micronutrient deficiencies and diet-related NCDs. If these guidelines are successfully implemented, health inequalities related to diet-associated diseases could be reduced. All three FBs provide useful information for the development of Estonian dietary guidelines for low income families, but further studies are needed to investigate which dietary changes are most acceptable.

## Figures and Tables

**Table 1:** Daily estimated energy requirements and recommended nutrient intakes for each member of the reference Estonian family of four [3,6].

	Mother		Father		Girl		Boy	
	Min	Max	Min	Max	Min	Max	Min	Max
Age (years)	35	39	40	44	15		13	
Weight (kg)	63.7		74.4		53.2		43.5	
PAL	1.6		1.6		1.73		1.73	
EER (kcal)	2050		2650		2350		2400	
EER (MJ)	8.58		11.1		9.83		10.0	
Protein (g)	51.3	102.5	66.3	132.5	58.8	117.5	60	120
Lipid total (g)	56.9	79.7	73.6	103.1	65.3	91.4	67	83.3
SFA (g)		22.8		29.4		26.1		26.7
MUFA (g)	22.7	45.6	29.4	58.9	26.1	52.2	27	53.3
PUFA (g)	11.4	22.8	14.7	29.4	13.1	26.1	13	26.7
ω-3 fatty acids (g)	2.27		2.9		2.61		2.67	
ω-6 fatty acids (g)	11.4		14.7		13.1		13.3	
TFA (g)		2.3		2.9		2.6		2.7
Cholesterol (mg)		300		300		300		300
Carbohydrates (g)	256	308	331	398	294	353	300	360
Fibre (g)	25		35		22		31.2	
Added total sugar (g)		51.3		66.3		58.8		60
Sodium (mg)		2400		2400		2400		2400
Potassium (mg)	3100	3700	3500	3700	3100		3300	
Calcium (mg)	800	2500	800	2500	900	2500	900	2500
Magnesium (mg)	320		380		320		300	
Iron (mg)	15	60	10	60	15	60	11	
Zinc (mg)	9	25	9	25	9	25	11	
Copper (mg)	0.9	5	0.9	5	0.9	5	0.7	
Selenium (μg)	50	300	60	300	50	300	40	
Phosphorus (mg)		3000		3000		3700		
Iodine (μg)	150	600	150	600	150	600	150	
Vitamin A (μg)	700		900		700		700	
Thiamine (mg)	1.1		1.4		1.2		1.2	
Riboflavin (mg)	1.3		1.7		1.4		1.4	
Vitamin B6 (mg)	1.5	25	1.8	25	1.5	25	1.8	25
Vitamin B12 (μg)	3		3		3		3	
Vitamin C (mg)	100	1000	100	1000	100	1000	70	
Vitamin D (μg)	10	100	10	100	10	100	10	100
Vitamin E (mg)	8	300	10	300	8	300	8	
Folate (μg)	300		300		330		270	
Niacin (mg)	15		19		16		16	

EER—Estimated Energy Requirement; PAL Physical activity level; SFA—saturated fatty acids; MUFA—mono-unsaturated fatty acids; PUFA—poly-unsaturated fatty acids; TFA—trans-fatty acids.

**Table 2:** Number of daily portions per food group as recommended in national dietary guidelines applied to the healthy food basket (HFB) and the nutritionally adequate and healthy food basket (NHFB) according to estimated energy requirements [6].

Food Categories (in bold) and Subcategories	Mother	Father	Girl and Boy
**Starchy foods: cereals and potatoes**	**7–9**	**10–14**	**8–12**
Breads	3–4	4–6	4–5
Pasta, rice, porridges, etc.	2–4	3–6	3–5
Potatoes	1–2	1–2	1–2
**Fruits, berries and vegetables**	**6–8**	**7–10**	**7–9**
Fruits and berries	2–3	3	3
Vegetables excl. potatoes	3–5	4–7	4–7
**Milk and dairy products**	**2–3**	**3**	**3**
**Fish, poultry, eggs, meat and meat products**	**3–4**	**3–4**	**3–4**
Fish, fish products and seafood	1–2	1–2	1–2
Meat and meat products, poultry and poultry products	1–2	1–2	1–2
Eggs	0.5	0.5	0.5
**Added fats, nuts, seeds and oleaginous fruits**	**6–7**	**9**	**9**
Nuts & Seeds	1–2	2–3	2–3
Oleaginous fruits, oils, fat spreads	5	6–7	6–7
**Sugar, sweets and savoury snacks**	**≤4**	**≤4**	**≤4**

**Table 3:** Abbreviations used for the models describing the different optimised food baskets by objective functions and constraints enforced.

Food Basket	Objective Function	Set of Constraints Enforced
Lowest cost FB (LCFB)	Cost (min)	EER, AMDR, RNIs
Nutritionally adequate FB (NFB)	TRD (min)	EER, AMDR, RNIs, MWFC
Health-promoting FB (HFB)	TRD (min)	EER, FBDG, MWFC (no macronutrient or micronutrient recommendations)
Nutritionally adequate, health-promoting FB (NHFB)	TRD (min)	EER, AMDR, RNIs, FBDG, MWFC

TRD—total relative deviation; EER—estimated energy requirements; AMDR, acceptable macronutrient distribution ranges; RNIs—recommended nutrient intakes; MWFC—minimum wage food cost.

**Table 4:** Composition of the lowest cost Food Basket (LCFB) fulfilling all recommended nutrient intakes for all members of an Estonian family, including weights and cost of the single foods.

Food Item (9)	Mother		Father		Girl		Boy		Family	
	Weight (g)	Cost (EUR)	Weight (g)	Cost (EUR)	Weight (g)	Cost (EUR)	Weight (g)	Cost (EUR)	Weight (g)	Cost (EUR)
Flour, wheat	153	0.10	189	0.12	249	0.16	157	0.10	748	0.49
Flour, rye	197	0.14	193	0.14	165	0.12	269	0.20	824	0.60
Flour, rye, wholegrain	0	0	96.1	0.08	0	0	17.3	0.01	113	0.09
Beans, broad	481	0.13	482	0.13	477	0.13	326	0.09	1766	0.46
Buttermilk	443	0.32	416	0.30	519	0.37	522	0.38	1900	1.37
Bream, hot-smoked	68.8	0.15	68.9	0.15	68.4	0.15	68.4	0.15	275	0.59
Liver, chicken	4.8	0.01	6.3	0.02	4.7	0.01	4.9	0.01	20.7	0.05
Oil, rapeseed	47.3	0.09	69.4	0.13	55.2	0.10	62.3	0.12	234	0.44
Salt, iodised	1.5	<0.01	1.4	<0.01	1.0	<0.01	0.8	<0.01	4.7	<0.01
TOTAL	1397	0.94	1522	1.07	1540	1.05	1428	1.05	5886	4.11
Average deviation % from reported intake	342		311		655		349		415	

**Table 5:** The weights, number of portions, and cost of food categories in the food baskets (FB) that are nutritionally adequate (NFB), include the Estonian food-based dietary guidelines (FBDG) only (HFB), and are both nutritionally adequate and include the Estonian FBDG (NHFB) per day compared with the Estonian National Dietary Survey (ENDS).

Food Category	Weight (g)				Number of Portions				Cost (EUR)		
	ENDS	NFB	HFB	NHFB	ENDS	NFB	HFB	NHFB	NFB	HFB	NHFB
Starchy food—cereals and potatoes	1064	2161	1600	2295	24.6	51.7	33.0	44.4	2.57	1.79	2.29
Fruits, vegetables and berries	1592	887	2140	2198	17.9	11.5	26.0	26.0	1.34	2.69	2.90
Milk and dairy products	1196	1389	1325	1351	7.9	10.9	11.5	11.4	2.26	2.37	2.13
Fish, poultry, eggs, meat and meat products	586	581	514	531	13.2	16.2	13.2	13.8	2.69	2.05	2.09
Added fats, nuts, seeds and other oleaginous fruits *	76	81	185	205	12.2	14.5	30.0	32.9	0.23	0.51	0.54
	(17)	(17)	(70)	(80)	(1.6)	(1.7)	(7.0)	(8.0)	(0.07)	(0.26)	(0.27)
Sugar, sweets and savoury snacks	2112	628	468	432	77.0	33.7	16.1	16.1	1.56	1.25	0.97
Salt	8.9	4.2	-	1.03	-	-	-	-	0.01	-	<0.01
TOTAL	6635	5732	6190	6913	153	138.4	130.0	145.0	10.66	10.66	10.92

* Values in parentheses indicate the shares attributable to oleaginous fruits.

**Table 6:** Major relative deviations of the intake of micronutrients, fats, sodium, and added sugars from Estonian dietary guidelines by family members in the health-promoting food basket (HFB).

	Mother	Father	Girl	Boy	Family
	**%**				
Vitamin D	66	74	79	72	73
Iodine	69	83	78	81	78
Iron	81	199	106	166	130
Folate	78	106	108	124	104
Calcium	87	113	98	97	99
Total lipids	108	114	114	122	115
Saturated fats	134	145	121	126	132
Total added sugars	128	86	323	128	107
Sodium	133	210	146	177	166

**Table 7:** Estonian food-based dietary guidelines for low SES families should include the following recommendations.

Eat more vegetables (such as cabbage, beetroot, onions); Eat more wholemeal and rye bread, cereals, and potato; Eat more pulses (such as peas and beans); Eat more fish and seafood (such as herring, mackerel, cod roe, and sprats); Eat more nuts and seeds; Use mainly (vitamin fortified) rapeseed oil rather than other oils and fats; Use mainly unsweetened and unflavoured dairy products; Eat less meat and poultry (fresh and especially, processed) and eat more liver, kidneys, and other offal; Eat less cheese; Eat less sugar, sweets, and savoury snacks; Eat less salt and buy iodised salt (and government to adopt universal salt iodisation into national legislation).

## Supplementary Materials

The following are available online at https://www.mdpi.com/2072-6643/12/9/2613/s1, Table S1: Nutritional composition of the simplest and lowest cost food basket (LCFB) for an Estonian family of four; Table S2: Composition of the nutritionally adequate food basket (NFB) for family of four per month; Table S3: Composition of the health-promoting food basket (HFB) for family of four per month; Table S4: Composition of the nutritionally adequate and health-promoting food basket NHFB for family of four per month.
