# Supplementary material for: Culturally Optimised Nutritionally Adequate Food Baskets for Dietary Guidelines for Minimum Wage Estonian Families
Source: Nutrients. 2020 Aug 27;12(9):2613. doi: 10.3390/nu12092613 (PMC7551125; doi:10.3390/nu12092613)
Supplement: Supplementary file 1 [file nutrients-12-02613-s001.zip › Lauk Estonian Food Baskets Table S4.docx]

**Table S4. Composition of the nutritionally adequate and health-promoting food basket NHFB for family of four per month.**

| **Category** | **Sub-category** | **Food item** | **Portions in FB** | **Weight edible (g)** | **Cost (€) per. Item** |
| --- | --- | --- | --- | --- | --- |
| Starchy foods: cereals and potatoes | Black bread, white bread, graham | Bread, rye | 299.6 | 8988 | 13.39 |
|  |  | Bread, graham | 167.8 | 5035 | 9.57 |
|  |  | Bread, white | 139.3 | 4179 | 5.12 |
|  | Rice, pasta, porridges etc. | Pasta, wholegrain | 31.5 | 2206 | 2.42 |
|  |  | Instant noodles, beef | 90.7 | 6352 | 7.22 |
|  |  | Rice, long grain, polished | 257.0 | 17992 | 10.97 |
|  |  | Buckwheat | 2.3 | 226 | 0.11 |
|  |  | Oats | 8.5 | 847 | 0.31 |
|  |  | Semolina | 4.9 | 486 | 0.12 |
|  |  | Flour, wheat | 13.5 | 338 | 0.22 |
|  |  | Flour, wheat, wholegrain | 19.6 | 491 | 0.48 |
|  |  | Flour, rye, wholegrain | 119.0 | 2975 | 2.35 |
|  |  | Corn flakes | 7.4 | 149 | 0.85 |
|  |  | Buffed wheat with honey | 30.3 | 607 | 2.77 |
|  |  | Potatoes, raw | 158.4 | 15843 | 13.83 |
|  |  | TOTAL PER. CATEGORY | 1350.0 | 66713 | 69.73 |
| Fruits and vegetables, berries | Fruits and berries | Apples, raw | 39.3 | 4712 | 7.80 |
|  |  | Bananas, raw | 65.7 | 3285 | 11.75 |
|  |  | Watermelon, raw | 25.6 | 3837 | 4.21 |
|  |  | Lemon, raw | 11.7 | 1525 | 5.06 |
|  |  | Lime, raw | 11.7 | 1525 | 4.25 |
|  |  | Nectarine, raw | 53.8 | 5377 | 9.40 |
|  |  | Apricot, raw | 6.1 | 614 | 1.25 |
|  |  | Rhubarb, raw | 3.7 | 558 | 1.03 |
|  |  | Black currant, frozen | 6.7 | 808 | 3.16 |
|  |  | Grapes, dried (raisins) | 22.8 | 456 | 2.55 |
|  |  | Tomato juice | 85.8 | 8577 | 8.15 |
|  | Vegetables excl. potatoes | Peas, dried | 39.1 | 1173 | 0.39 |
|  |  | Beans, red | 17.4 | 521 | 0.56 |
|  |  | Cabbage, raw | 86.9 | 8693 | 3.57 |
|  |  | Chinese cabbage, raw | 20.0 | 2001 | 2.93 |
|  |  | Zucchini, raw | 12.7 | 1016 | 1.21 |
|  |  | Onion, yellow, raw | 77.6 | 7761 | 2.50 |
|  |  | Onion, red, raw | 2.3 | 231 | 0.25 |
|  |  | Tomatoes, chopped, canned | 34.0 | 2043 | 3.30 |
|  |  | Celery, fresh | 40.8 | 2043 | 4.07 |
|  |  | Beetroot, raw | 97.0 | 7761 | 7.47 |
|  |  | Beetroot, boiled | 26.7 | 2140 | 2.85 |
|  |  | Champignon, raw | 1.3 | 174 | 0.59 |
|  |  | TOTAL PER. CATEGORY | 788.9 | 66829 | 88.30 |

**Table S4. Continued**

| **Category** | **Sub-category** | **Food item** | **Portions in FB** | **Weight edible (g)** | **Cost (€) per. Item** |
| --- | --- | --- | --- | --- | --- |
| Milk and dairy products |  | Milk, whole | 174.4 | 29655 | 26.54 |
|  |  | Buttermilk | 6.1 | 1212 | 0.87 |
|  |  | Yogurt, natural | 2.1 | 317 | 0.71 |
|  |  | Sour cream, 20% fat | 7.8 | 390 | 0.84 |
|  |  | Cream, 10% fat | 7.7 | 769 | 1.40 |
|  |  | Condensed milk without sugar | 74.3 | 2229 | 8.29 |
|  |  | Curd, plain, light (Quark) | 10.3 | 1338 | 4.95 |
|  |  | Yogurt, 1-2% fat, with fruits | 17.9 | 2501 | 7.25 |
|  |  | Yogurt, 4-6% fat, with fruits | 5.8 | 578 | 1.75 |
|  |  | Curd, creamed, with fruits (Quark) | 13.9 | 1042 | 4.74 |
|  |  | Cheese, Edam | 1.2 | 42 | 0.34 |
|  |  | Cheese, Emmental | 10.3 | 258 | 2.35 |
|  |  | Cheese spread, plain | 15.1 | 753 | 4.82 |
|  |  | TOTAL PER. CATEGORY | 346.8 | 41083 | 64.85 |
| Fish, poultry, eggs, meat and meat products | Fish and fish products | Bream, hot-smoked | 23.4 | 1405 | 3.04 |
|  |  | Mackerel, cold-smoked | 26.7 | 800 | 5.64 |
|  |  | Sprat, smoked, in oil | 48.5 | 1455 | 9.64 |
|  |  | Sprat, smoked, pate | 44.6 | 1339 | 5.10 |
|  |  | Mussels, blue | 3.9 | 174 | 2.67 |
|  | Meat and meat products, poultry and poultry products | Chicken, whole leg | 72.4 | 1811 | 8.62 |
|  |  | Picnic Shoulder | 3.2 | 112 | 0.82 |
|  |  | Minced pork meat | 11.2 | 335 | 2.10 |
|  |  | Weiner, chicken | 7.1 | 498 | 1.48 |
|  |  | Meatballs, frozen | 42.6 | 1278 | 5.50 |
|  |  | Chicken, liver | 4.0 | 200 | 0.48 |
|  |  | Chicken, neck, fresh | 11.7 | 584 | 2.25 |
|  |  | Liver, pork | 58.2 | 2617 | 6.37 |
|  |  | Dumplings (pork & beef) | 2.5 | 197 | 0.77 |
|  |  | Eggs, Chicken | 60.8 | 3344 | 8.94 |
|  |  | TOTAL PER. CATEGORY | 420.7 | 16147 | 63.42 |
| Added oils and fats, nuts, seeds & oilseeds | Nuts & seeds | Peanuts | 91.2 | 912 | 3.60 |
|  |  | Linseed | 152.0 | 1520 | 4.53 |
|  | Oilseeds, oils, fat spreads | Lard | 106.8 | 534 | 1.66 |
|  |  | Margarine, for cooking | 59.1 | 295 | 0.82 |
|  |  | Rapeseed oil | 584.9 | 2925 | 5.53 |
|  |  | Sunflower oil | 7.3 | 37 | 0.08 |
|  |  | TOTAL PER. CATEGORY | 1001.3 | 6223 | 16.22 |

Table S4. Continued

| **Category** | **Sub-category** | **Food item** | **Portions in FB** | **Weight edible (g)** | **Cost (€) per. Item** |
| --- | --- | --- | --- | --- | --- |
| Sugar, sweet and savoury snacks, alcoholic drinks |  | Sugar, white | 24.8 | 248 | 0.67 |
|  |  | Sugar, brown | 100.2 | 1002 | 2.66 |
|  |  | Honey | 26.5 | 265 | 2.82 |
|  |  | Chocolate, milk | 8.4 | 84 | 1.08 |
|  |  | Strawberry, jam | 31.1 | 621 | 3.64 |
|  |  | Ice cream, cream, chocolate | 20.6 | 413 | 3.42 |
|  |  | Condensed milk with sugar | 165.4 | 1654 | 6.63 |
|  |  | Multi juice drink | 1.6 | 157 | 0.18 |
|  |  | Plum nectar | 29.8 | 2979 | 2.96 |
|  |  | Peaches, in syrup | 1.3 | 130 | 0.46 |
|  |  | Plums, in syrup | 0.2 | 17 | 0.13 |
|  |  | Cookies, plain | 10.1 | 51 | 0.16 |
|  |  | Water, carbonated, lemon flavour | 49.9 | 4990 | 3.32 |
|  |  | Popcorn, plain | 9.3 | 65 | 0.39 |
|  |  | Mayonnaise | 7.4 | 186 | 0.52 |
|  |  | Caesar dressing | 0.7 | 17 | 0.09 |
|  |  | Tomato soup, dry, instant | 0.1 | 40 | 0.05 |
|  |  | Chicken soup, dry, instant | 0.5 | 166 | 0.07 |
|  |  | Beer, less than 6% alcohol | 0.1 | 42 | 0.14 |
|  |  | TOTAL PER. CATEGORY | 487.9 | 13125 | 29.40 |
|  |  | Salt, iodized | 0.0 | 31 | 0.06 |
|  |  | **TOTAL MONTHLY BASKET** | **4396** | **210151** | **331.97** |
