# Supplementary material for: Culturally Optimised Nutritionally Adequate Food Baskets for Dietary Guidelines for Minimum Wage Estonian Families
Source: Nutrients. 2020 Aug 27;12(9):2613. doi: 10.3390/nu12092613 (PMC7551125; doi:10.3390/nu12092613)
Supplement: Supplementary file 1 [file nutrients-12-02613-s001.zip › Lauk Estonian Food Baskets Table S1.docx]

**Table S1. Nutritional composition of the simplest and lowest-cost daily food basket (LCFB) for an Estonian family of four.**

| **Food Item** | **Amount (g)** | **Price (€)** | **Energy, kcal** | **Protein (g)** | **Lipids, total (g)** | **Saturated fatty acids (g)** | **FA Mono (g)** | **FA Poly (g)** | **n-3 poly (g)** | **n-6poly(g)** | **Trans FAs (g)** | **Cholesterol (mg)** | **Carbohydrates (g)** | **Fibre (g)** | **Added Tot Sugars (g)** | **Sodium (mg)** | **Potassium (mg)** | **Calcium (mg)** |
| --- | --- | --- | --- | --- | --- | --- | --- | --- | --- | --- | --- | --- | --- | --- | --- | --- | --- | --- |
| \| Flour, wheat \| \| --- \| | 748 | 0.49 | 2365 | 74.0 | 12.7 | 1.4 | 1.8 | 5.3 | 0.3 | 1.5 | <0.1 | 0 | 502 | 26.2 | 3.0 | 3.0 | 1121 | 97.2 |
| Flour, rye | 824 | 0.60 | 2497 | 82.4 | 19.0 | 2.5 | 1.9 | 9.5 | 0.6 | 4.9 | <0.1 | 0 | 570 | 141 | 19.8 | 8.2 | 4121 | 247 |
| Flour, rye, wholegrain | 113 | 0.09 | 366 | 12.2 | 1.7 | 0.2 | 0.2 | 1.1 | 0.2 | 1.1 | <0.1 | 0 | 83.3 | 16.0 | 2.2 | 2.3 | 567 | 37.4 |
| Beans, broad | 1766 | 0.46 | 1095 | 84.8 | 8.8 | 2.5 | 0.4 | 5.4 | 0.0 | 2.9 | <0.1 | 0 | 178 | 63.6 | 14.1 | 724 | 3408 | 31.8 |
| Buttermilk | 1901 | 1.37 | 648 | 57.0 | 9.5 | 5.7 | 1.9 | <0.1 | 0.6 | <0.1 | <0.1 | 114 | 83.6 | <0.1 | 83.6 | 855 | 3231 | 2471 |
| Bream, hot-smoked | 275 | 0.59 | 340 | 58.5 | 11.6 | 2.0 | 5.0 | 3.4 | 2.7 | 0.5 | <0.1 | 232 | 0.0 | <0.1 | 0 | 2270 | 1184 | 221 |
| Chicken, liver | 20.7 | 0.05 | 31.7 | 4.9 | 0.9 | 0.3 | 0.2 | 0.2 | 0.0 | 0.2 | <0.1 | 136 | 1.1 | <0.1 | 0 | 19.0 | 99.3 | 1.7 |
| Rapeseed oil | 234 | 0.44 | 2109 | 0.0 | 234 | 16.4 | 148 | 70.3 | 26.0 | 51.8 | <0.1 | <0.1 | 0 | 0 | 0 | 0 | 0 | 0 |
| Salt, iodized | 4.71 | 0.01 | 0 | 0 | 0 | 0 | 0 | 0 | 0 | 0 | 0 | 0 | 0 | 0 | 0 | 1209 | 1.7 | 7.1 |
| **SUM** | **5 886** | **€4.11** | **9 450** | **374** | **298** | **31.0** | **159** | **95.2** | **30.4** | **63.0** | **<0.1** | **481** | **1 417** | **247** | **123** | **5 090** | **13 732** | **3 400** |
| **% of critical value** |  |  | **100** | **158** | **114** | **30** | **151** | **182** | **291** | **120** | **0** | **40** | **120** | **218** | **52** | **53** | **106** | **100** |

| **Food Item** | **Magnesium (mg)** | **Iron (mg)** | **Zinc (mg)** | **Copper (mg)** | **Manganese (mg)** | **Selenium (µg)** | **Phosphorus (mg)** | **Iodine (µg)** | **Vit A RAE** | **Thiamin (mg)** | **Riboflavin (mg)** | **Vit B6 (mg)** | **Vit B12 (µg)** | **Vit C (mg)** | **Vit D µg** | **Vit E (mg)** | **Folate Tot (µg)** | **Niacin_(mg)** |
| --- | --- | --- | --- | --- | --- | --- | --- | --- | --- | --- | --- | --- | --- | --- | --- | --- | --- | --- |
| \| Flour, wheat \| \| --- \| | 157 | 5.2 | 6.0 | 1.5 | 3.5 | 74.8 | 748 | 15.0 | 0.0 | 3.2 | 0.4 | 0.6 | 0.0 | 0.0 | 0.0 | 2.4 | 135 | 6.7 |
| Flour, rye | 907 | 40.4 | 27.2 | 4.8 | 30.5 | 42.9 | 2967 | 41.2 | 4.4 | 2.5 | 1.1 | 2.9 | 0.0 | 0.0 | 0.0 | 13.2 | 569 | 8.2 |
| Flour, rye, wholegrain | 105 | 4.5 | 4.2 | 0.4 | 3.2 | 1.7 | 404 | 5.7 | 0.6 | 0.4 | 0.2 | 0.3 | 0.0 | 0.0 | 0.0 | 1.1 | 88.4 | 2.2 |
| Beans, broad | 547 | 26.5 | 8.3 | 1.1 | 4.6 | 17.7 | 1289 | 7.1 | 247 | 2.3 | 1.6 | 0.5 | 0.0 | 350 | 0.0 | 0.7 | 1024 | 21.2 |
| Buttermilk | 228 | 1.9 | 9.5 | 0.0 | 0.0 | 28.5 | 1901 | 266 | 64.6 | 0.6 | 3.8 | 1.0 | 6.5 | 19.0 | 1.5 | 0.2 | 55.1 | 1.9 |
| Bream, hot-smoked | 89.6 | 1.4 | 1.6 | 0.1 | 0.2 | 85.1 | 713 | 161 | 7.4 | 0.2 | 0.2 | 1.2 | 5.5 | 0.0 | 38.4 | 5.5 | 16.5 | 26.5 |
| Chicken, liver | 5.8 | 2.3 | 1.2 | 0.1 | 0.1 | 12.7 | 108 | 0.7 | 2675 | <0.1 | 0.3 | 0.2 | 8.8 | 1.4 | 0.1 | 0.1 | 166 | 2.3 |
| Rapeseed oil | 0.0 | 0.2 | <0.1 | <0.1 | <0.1 | <0.1 | <0.1 | 0 | 0.6 | 0 | 0 | 0 | 0 | 0 | 0 | 42.2 | 0 | 0 |
| Salt, iodized | 1.6 | 0.0 | 0.0 | 0.0 | 0.0 | 0.0 | 3.3 | 104 | 0 | 0 | 0 | 0 | 0 | 0 | 0 | 0 | 0 | 0 |
| **SUM** | **2 041** | **82.5** | **58.0** | **7.99** | **42.1** | **263** | **8 131** | **600** | **3 000** | **9.1** | **7.6** | **6.6** | **20.8** | **370** | **40.0** | **65.4** | **2 053** | **69** |
| **% of critical value** | **155%** | **162%** | **153%** | **235%** |  | **132%** | **119%** | **100%** | **100%** | **186%** | **131%** | **100%** | **173%** | **100%** | **100%** | **192%** | **171%** | **105%** |
