# Supplementary material for: Culturally Optimised Nutritionally Adequate Food Baskets for Dietary Guidelines for Minimum Wage Estonian Families
Source: Nutrients. 2020 Aug 27;12(9):2613. doi: 10.3390/nu12092613 (PMC7551125; doi:10.3390/nu12092613)
Supplement: Supplementary file 1 [file nutrients-12-02613-s001.zip › Lauk Estonian Food Baskets Table S2.docx]

**Table S2. Composition of the nutritionally adequate food basket (NFB) for family of four per month.**

| **Category** | **Sub-category** | **Food item** | **Portions in FB** | **Weight (g) (edible)** | **Cost (€) per. Item** |
| --- | --- | --- | --- | --- | --- |
| Starchy foods: cereals and potatoes | Black bread, white bread, graham | Bread, rye | 155 | 4651 | 6.93 |
|  |  | Bread, graham | 25.5 | 764 | 1.45 |
|  |  | Bread, white | 263 | 7885 | 9.66 |
|  | Rice, pasta, porridges etc. | Pasta, wheat | 78.7 | 5510 | 4.95 |
|  |  | Pasta, wholegrain | 55.3 | 3871 | 4.24 |
|  |  | Instant noodles, beef | 71.1 | 4980 | 5.66 |
|  |  | Buckwheat | 2.3 | 226 | 0.11 |
|  |  | Oats | 13.3 | 1333 | 0.49 |
|  |  | Flour, wheat | 95.5 | 2388 | 1.58 |
|  |  | Flour, rye, wholegrain | 347 | 8686 | 6.86 |
|  |  | Flour, oat, wholegrain | 228 | 5708 | 9.08 |
|  |  | Corn flakes | 47.6 | 951 | 5.45 |
|  |  | Fruit muesli | 1.5 | 30 | 0.17 |
|  | Potatoes | Potatoes, raw | 158 | 15843 | 13.83 |
|  |  | Potatoes, frozen | 28.7 | 2871 | 7.77 |
|  |  | TOTAL PER. CATEGORY | 15726 | 65697 | 78.23 |
| Fruits and vegetables, berries | Fruits and berries | Apples, raw | 7.0 | 845 | 1.40 |
|  |  | Watermelon, raw | 5.6 | 845 | 0.93 |
|  |  | Nectarine, raw | 8.4 | 845 | 1.48 |
|  |  | Rhubarb, raw | 2.5 | 369 | 0.68 |
|  |  | Strawberries, frozen | 3.4 | 408 | 1.89 |
|  |  | Black currant, frozen | 6.7 | 808 | 3.16 |
|  |  | Bananas, dried | 26.3 | 526 | 3.63 |
|  |  | Grapes, dried (raisins) | 18.7 | 373 | 2.09 |
|  | Vegetables excl. potatoes | Tomato juice | 73.4 | 7339 | 6.97 |
|  |  | Peas, dried | 15.2 | 456 | 0.15 |
|  |  | Beans, red | 2.0 | 61 | 0.07 |
|  |  | Cabbage, raw | 36.7 | 3667 | 1.51 |
|  |  | Chinese cabbage, raw | 13.5 | 1353 | 1.98 |
|  |  | Zucchini, raw | 0.8 | 65 | 0.08 |
| Fruits and vegetables, berries | Vegetables excl. potatoes | Onion, yellow, raw | 31.6 | 3156 | 1.02 |
|  |  | Garlic, raw | 38.2 | 764 | 5.66 |
|  |  | Spinach, frozen | 7.1 | 887 | 1.81 |
|  |  | Beetroot, raw | 39.4 | 3156 | 3.04 |
|  |  | Beetroot, boiled | 10.6 | 847 | 1.13 |
|  |  | Champignon, raw | 0.2 | 22 | 0.07 |
|  |  | Champignon, frozen | 0.7 | 87 | 0.33 |
|  |  | Chanterelle, frozen | 0.7 | 91 | 1.67 |
|  |  | TOTAL PER. CATEGORY | 349 | 26968 | 40.73 |

**Table S2. Continued**

| **Category** | **Sub-category** | **Food item** | **Portions in FB** | **Weight (g) (edible)** | **Cost (€) per. Item** |
| --- | --- | --- | --- | --- | --- |
| Milk and dairy products |  | Milk, whole | 186.2 | 31653 | 28.33 |
|  |  | Buttermilk | 3.0 | 599 | 0.43 |
|  |  | Yogurt, natural | 3.1 | 460 | 1.03 |
|  |  | Sour cream, 20% fat | 9.0 | 452 | 0.97 |
|  |  | Cream, 10% fat | 7.8 | 775 | 1.41 |
|  |  | Condensed milk without sugar | 24.5 | 736 | 2.74 |
|  |  | Curd, plain (Quark) | 1.7 | 222 | 0.99 |
|  |  | Curd, plain, light (Quark) | 10.3 | 1338 | 4.95 |
|  |  | Yogurt, 2% fat, fruit taste, with sugar | 22.0 | 3079 | 8.93 |
|  |  | Curd, creamed, fruit taste, with sugar (Quark) | 13.9 | 1042 | 4.74 |
|  |  | Cottage cheese, with sour cream | 3.9 | 295 | 1.24 |
|  |  | Cheese, Edam | 12.2 | 427 | 3.40 |
|  |  | Cheese, Havarti | 0.7 | 23 | 0.19 |
|  |  | Cheese, Emmental | 18.6 | 466 | 4.25 |
|  |  | Cheese, Parmesan | 2.3 | 82 | 1.45 |
|  |  | Cheese spread, plain | 11.5 | 573 | 3.67 |
|  |  | TOTAL PER. CATEGORY | 331 | 42220 | 68.72 |
| Fish, poultry, eggs, meat and meat products | Fish, fish products and seafood | Bream, hot-smoked | 14.7 | 882 | 1.91 |
|  |  | Mackerel, cold-smoked | 34.4 | 1033 | 7.29 |
|  |  | Sprat, smoked, in oil | 42.1 | 1263 | 8.37 |
|  |  | Sprat, smoked, paté | 38.7 | 1161 | 4.42 |
|  |  | Mussels, blue | 3.9 | 174 | 2.67 |
|  |  | Fish roe, red/black | 2.0 | 61 | 1.31 |
|  | Meat and meat products, poultry and poultry products | Chicken, whole leg | 71.9 | 1798 | 8.56 |
|  |  | Chicken, wings | 15.1 | 378 | 2.68 |
|  |  | Pork shoulder | 14.8 | 520 | 3.82 |
|  |  | Bacon | 22.7 | 227 | 3.00 |
|  |  | Minced pork meat | 74.7 | 2240 | 14.05 |
|  |  | Weiner, chicken | 24.2 | 1694 | 5.05 |
|  |  | Meatballs, frozen | 19.4 | 582 | 2.50 |
|  |  | Chicken, liver | 5.4 | 270 | 0.64 |
|  |  | Chicken, neck, fresh | 26.4 | 1319 | 5.08 |
|  |  | Liver, pork | 39.2 | 1764 | 4.29 |
|  |  | Eggs, Chicken | 42.0 | 2310 | 6.18 |
|  |  | TOTAL PER. CATEGORY | 492 | 17674 | 81.80 |
| Added fats, nuts, seeds, and oleaginous fruits | Nuts and seeds | Peanuts | 11.9 | 119 | 0.47 |
|  |  | Sunflower seeds, roasted, w/o salt | 18.1 | 181 | 1.08 |
|  |  | Linseed | 22.5 | 225 | 0.67 |
|  | Oils, fat spreads | Lard | 126.8 | 634 | 1.97 |
|  |  | Margarine, for cooking | 75.6 | 378 | 1.05 |
|  |  | Rapeseed oil | 148.3 | 742 | 1.40 |
|  |  | Sunflower oil | 36.5 | 183 | 0.40 |
|  |  | TOTAL PER. CATEGORY | 440 | 2462 | 7.03 |

**Table S2. Continued**

| **Category** | **Sub-category** | **Food item** | **Portions in FB** | **Weight (g) (edible)** | **Cost (€) per. Item** |
| --- | --- | --- | --- | --- | --- |
| Sugar, sweets and savoury snacks |  | Sugar, brown | 66.5 | 665 | 1.76 |
|  |  | Honey | 33.0 | 330 | 3.52 |
|  |  | Chocolate, milk | 25.6 | 256 | 3.31 |
|  |  | Strawberry, jam | 36.0 | 721 | 4.22 |
|  |  | Ice cream, cream, vanilla | 16.3 | 326 | 2.57 |
|  |  | Ice cream, cream, chocolate | 4.3 | 87 | 0.72 |
|  |  | Condensed milk with sugar | 97.3 | 973 | 3.90 |
|  |  | Multi juice drink | 40.3 | 4026 | 4.71 |
|  |  | Peaches, in syrup | 1.3 | 130 | 0.46 |
|  |  | Plums, in syrup | 0.2 | 22 | 0.17 |
|  |  | Cookies, plain | 485 | 2422 | 7.84 |
|  |  | Coca Cola, zero | 19.5 | 1954 | 1.93 |
|  |  | Water, carbonated, lemon flavour | 49.9 | 4990 | 3.32 |
|  |  | Potato chips, sour cream and onion | 16.1 | 113 | 1.12 |
|  |  | Popcorn, plain | 33.5 | 235 | 1.40 |
|  |  | Mayonnaise | 13.9 | 346 | 0.97 |
|  |  | Caesar dressing | 8.9 | 223 | 1.16 |
|  |  | Beer, less than 6% alcohol | 3.2 | 1286 | 4.24 |
|  |  | TOTAL PER. CATEGORY | 950 | 19104 | 47.31 |
|  |  | Salt, iodized |  | 129 | 0.23 |
|  | **TOTAL MONTHLY BASKET** | | **4133** | 174253 | **324.06** |
