# Supplementary material for: Culturally Optimised Nutritionally Adequate Food Baskets for Dietary Guidelines for Minimum Wage Estonian Families
Source: Nutrients. 2020 Aug 27;12(9):2613. doi: 10.3390/nu12092613 (PMC7551125; doi:10.3390/nu12092613)
Supplement: Supplementary file 1 [file nutrients-12-02613-s001.zip › Lauk Estonian Food Baskets Table S3.docx]

**Table S3. Composition of the health-promoting food basket (HFB) for family of four per month.**

| **Category** | **Sub-category** | **Food item** | **Portions in FB** | **Weight (g) (edible)** | **Cost (€) per. item** |
| --- | --- | --- | --- | --- | --- |
| Starchy foods: cereals and potatoes | Black bread, white bread, graham bread | Bread, rye | 272 | 8173 | 12.18 |
|  |  | Bread, white | 184 | 5507 | 6.75 |
|  | Rice, pasta, porridges etc. | Instant noodles, beef | 213 | 14883 | 16.92 |
|  |  | Couscous | 3.1 | 156 | 0.18 |
|  |  | Semolina | 14.0 | 1403 | 0.35 |
|  |  | Flour, wheat | 140 | 3504 | 2.31 |
|  |  | Buffed wheat with honey | 49.1 | 981 | 4.49 |
|  |  | Potatoes, raw | 128 | 12803 | 11.18 |
|  |  | TOTAL PER. CATEGORY | 1003 | 47410 | 54.34 |
| Fruits and vegetables, berries | Fruits and berries | Apples, raw | 36.9 | 4428 | 7.33 |
|  |  | Bananas, raw | 54.0 | 2702 | 9.67 |
|  |  | Watermelon, raw | 41.2 | 6187 | 6.78 |
|  |  | Nectarine, raw | 61.9 | 6187 | 10.81 |
|  |  | Apricot, raw | 11.4 | 1144 | 2.32 |
|  |  | Grapes, yellow/green, raw | 10.2 | 613 | 2.23 |
|  |  | Black currant, frozen | 10.1 | 1216 | 4.75 |
|  |  | Grapes, dried (raisins) | 33.7 | 673 | 3.77 |
|  |  | Tomato juice | 73.4 | 7339 | 6.97 |
|  | Vegetables excl. potatoes | Peas, dried | 52.1 | 1563 | 0.52 |
|  |  | Cabbage, raw | 94.9 | 9494 | 3.90 |
|  |  | Onion, yellow, raw | 95.4 | 9545 | 3.08 |
|  |  | Tomatoes, chopped, canned | 43.8 | 2631 | 4.25 |
|  |  | Celery, fresh | 77.3 | 3864 | 7.71 |
|  |  | Beetroot, raw | 90.8 | 7266 | 6.99 |
|  |  | Champignon, raw | 1.5 | 200 | 0.67 |
|  |  | TOTAL PER. CATEGORY | 789 | 65052 | 81.76 |
| Milk and dairy products |  | Milk, 2.5% fat | 11.8 | 2364 | 1.87 |
|  |  | Milk, whole | 155 | 26329 | 23.56 |
|  |  | Yogurt, natural | 2.9 | 430 | 0.96 |
|  |  | Yogurt, Greek, plain | 0.3 | 30 | 0.10 |
|  |  | Sour cream, 20% fat | 22.2 | 1112 | 2.39 |
|  |  | Cream, 35% fat | 28.4 | 851 | 3.28 |
|  |  | Curd, plain, light (Quark) | 4.5 | 582 | 2.15 |
|  |  | Curd, creamed (Quark) | 8.1 | 977 | 3.55 |
|  |  | Yogurt, 2-4% fat, with fruits | 9.2 | 1294 | 3.68 |
|  |  | Yogurt, 4-6% fat, with fruits | 29.1 | 2905 | 8.80 |
|  |  | Cottage cheese, with sour cream | 17.8 | 1338 | 5.63 |
|  |  | Cheese, Havarti | 14.7 | 513 | 4.25 |
|  |  | Cheese, Emmental | 28.9 | 723 | 6.60 |
|  |  | Cheese spread, plain | 16.3 | 817 | 5.23 |
|  |  | TOTAL PER. CATEGORY | 349 | 40266 | 72.06 |

**Table S3. Continued**

|  |  |  |  |  |  |
| --- | --- | --- | --- | --- | --- |
| **Category** | **Sub-category 1** | **Food item** | **Portions in FB** | **Weight (g) (edible)** | **Cost (€) per. item** |
| Fish, poultry, eggs, meat and meat products | Fish and fish products | Bream, hot-smoked | 2.2 | 133 | 0.29 |
|  |  | Herring, Atlantic, salted | 34.2 | 1025 | 5.19 |
|  |  | Mackerel, cold-smoked | 10.9 | 328 | 2.32 |
|  |  | Sprat, smoked, in oil | 33.7 | 1011 | 6.70 |
|  |  | Sprat, smoked, pate | 34.2 | 1025 | 3.90 |
|  |  | Mussels, blue | 2.9 | 130 | 2.00 |
|  |  | Fish roe | 3.5 | 104 | 2.24 |
|  | Meat and meat products, poultry and poultry products | Chicken, whole leg | 87.0 | 2176 | 10.36 |
|  |  | Picnic Shoulder | 5.1 | 178 | 1.31 |
|  |  | Minced pork meat | 17.8 | 533 | 3.34 |
|  |  | Wiener, chicken | 32.5 | 2276 | 6.78 |
|  |  | Chicken, liver | 8.3 | 415 | 0.99 |
|  |  | Liver, pork | 48.0 | 2158 | 5.25 |
|  |  | Liver sausage | 19.5 | 780 | 2.83 |
|  |  | Eggs, Chicken | 60.8 | 3344 | 8.94 |
|  |  | TOTAL PER. CATEGORY | 401 | 15617 | 62.44 |
| Added oils and fats, nuts, seeds & oilseeds | Nuts & seeds | Peanuts | 160 | 1596 | 6.30 |
|  |  | Linseed | 53.2 | 532 | 1.59 |
|  | Oilseeds, oils, fat spreads | Lard | 127 | 634 | 1.97 |
|  |  | Margarine, for cooking | 75.6 | 378 | 1.05 |
|  |  | Rapeseed oil | 497 | 2484 | 4.69 |
|  |  | TOTAL PER. CATEGORY | 912 | 5624 | 15.60 |
| Sugar, sweet, savoury snacks and alcoholic drinks |  | Sugar, brown | 148 | 1475 | 3.91 |
|  |  | Honey | 33.0 | 330 | 3.52 |
|  |  | Chocolate, milk | 36.7 | 367 | 4.74 |
|  |  | Strawberry, jam | 36.0 | 721 | 4.22 |
|  |  | Ice cream, cream, vanilla | 3.6 | 72 | 0.57 |
|  |  | Ice cream, cream, chocolate | 26.4 | 529 | 4.38 |
|  |  | Condensed milk with sugar | 70.4 | 704 | 2.82 |
|  |  | Plum nectar | 40.3 | 4026 | 4.01 |
|  |  | Peaches, in syrup | 1.5 | 152 | 0.53 |
|  |  | Water, carbonated, lemon flavour | 36.4 | 3639 | 2.42 |
|  |  | Popcorn, plain | 49.6 | 347 | 2.07 |
|  |  | Mayonnaise | 1.6 | 41 | 0.11 |
|  |  | Tomato soup, dry, instant | 0.3 | 105 | 0.14 |
|  |  | Chicken soup, dry, instant | 1.2 | 423 | 0.18 |
|  |  | Beer, less than 6% alcohol | 3.2 | 1285 | 4.24 |
|  |  | TOTAL PER. CATEGORY | 488 | 14217 | 37.87 |
|  |  | **TOTAL MONTHLY BASKET** | **3 942** | **188186** | **324.06** |
